# Jasmonic acid and glucose synergistically modulate the accumulation of glucosinolates in *Arabidopsis thaliana*

**DOI:** 10.1093/jxb/ert348

**Published:** 2013-10-22

**Authors:** Rongfang Guo, Wangshu Shen, Hongmei Qian, Min Zhang, Lihong Liu, Qiaomei Wang

**Affiliations:** ^1^Department of Horticulture, Zhejiang University, Hangzhou 310058, China; ^2^Key Laboratory of Horticultural Plant Growth, Development and Quality Improvement, Ministry of Agriculture, Hangzhou 310058, China

**Keywords:** ABI5, *Arabidopsis thaliana*, glucose, glucosinolate, jasmonic acid, salicylic acid.

## Abstract

The interplay of plant hormones and glucose (Glu) in regulating glucosinolate accumulation in *Arabidopsis thaliana* was investigated in this study. Glucose-induced glucosinolate biosynthesis was enhanced significantly by the addition of jasmonic acid (JA), whereas the synergistic effect of salicylic acid (SA) and Glu was less obvious. The enhanced glucosinolate accumulation is associated with elevated expression of genes in glucosinolate biosynthetic pathway, as well as the transcription factors involved in their regulation, such as *MYB28*, *MYB29*, *MYB34*, and *MYB122*. The induction of indolic and aliphatic glucosinolates after treatment with JA and Glu in JA-insensitive mutants, *coi1*, *jar1*, and *jin1*, was compromised. Moreover, the effect of JA and Glu on glucosinolate contents was dramatically reduced in Glu-insensitive mutants, *rgs1-2* and *abi5-7*. These results indicate a crosstalk between JA and Glu signalling in the regulation of glucosinolate biosynthesis. JA signalling, RGS1 (the putative membrane receptor of Glu signalling), and ABI5, are involved in the synergistic effect of JA and Glu on glucosinolate accumulation.

## Introduction

Glucosinolates are a group of secondary metabolites containing nitrogen and sulphur elements. They exist mainly in plants of the order Brassicales (Bones and Rossiter, 1996). Glucosinolates and their hydrolysis metabolites are involved in diverse functions such as flavour (Baik *et al.*, 2003), chemoprotection (Bradburne and Mithen, 2000; Mithen *et al.*, 2000; Clay *et al.*, 2009), antioxidant activity (Keck and Finley, 2004), and resistance to biotic and abiotic stresses (Brader *et al.*, 2006; Hopkins *et al.*, 2009; Hirayama and Shinozaki, 2010). The biosynthetic pathways of glucosinolates have been elucidated in *Arabidopsis* using biochemical and genetic approaches (Du *et al.*, 1995; Du and Halkier, 1996; Halkier and Du, 1997; Chen and Andreasson, 2001; Grubb and Abel, 2006; Halkier and Gershenzon, 2006). Indolic glucosinolates are derived from tryptophan, aromatic glucosinolates from phenylalanine, and aliphatic glucosinolates mainly from methionine. In shoots of *Arabidopsis thaliana*, only indolic and aliphatic glucosinolates are present. In the biosynthesis of glucosinolate core structure, substrates tryptophan and methionine are first metabolized by CYP79B2/CYP79B3 and CYP79F1/CYP79F2 (Chen *et al.*, 2003) to the corresponding aldoximes, respectively. Aldoximes are further catalysed by CYP83B1 and CYP83A1 to form S-alkylthiohydroximates (Bak and Feyereisen, 2001). After cleaved by C-S lyase, S-alkylthiohydroximates are converted into thiohydroximates (Mikkelsen *et al.*, 2004). Then the thiohydroximates are glycosylated by UDP glycosyltransferases to desulphoglucosinolates (Gachon *et al.*, 2005), followed by the sulphation by sulphotransferases (Piotrowski *et al.*, 2004). Six related members of the R2R3 MYB transcription factor family within *Arabidopsis* are involved in regulation of glucosinolate biosynthesis. MYB28, MYB29, and MYB76 are commonly defined as regulators of aliphatic glucosinolate biosynthesis (Gigolashvili *et al.*, 2007, 2008), whereas MYB34, MYB51, and MYB122 regulate indolic glucosinolate biosynthesis (Celenza *et al.*, 2005; Gigolashvili *et al.*, 2007, 2008).

Plants are able to integrate a wide variety of stimuli from both internal and environmental sources to alter their metabolic activities. Glucosinolate metabolism has evolved as a result of plant–environment interactions. Glucosinolate biosynthesis is regulated by plant hormones such as jasmonic acid (JA) and salicylic acid (SA) (Kiddle *et al.*, 1994; Doughty *et al.*, 1995; Brader *et al.*, 2001; Farnham *et al.*, 2004; Yan and Chen, 2007). Jasmonates positively regulate glucosinolate synthesis in various plant species and tissues by activating the expression of transcription factors (*MYB34*, and *MYB51*) and biosynthetic genes (*CYP79B2*, *CYP79B3*, *CYP79F1*, and *CYP79F2*) involved in glucosinolate biosynthesis (Doughty *et al.*, 1995; Brader *et al.*, 2001; Mikkelsen *et al.*, 2003; Dombrecht *et al.*, 2007). SA exerts a complicated effect on inducing the accumulation of glucosinolates. Kiddle *et al.* (1994) reported that SA specifically induced nearly all glucosinolates, especially 2-phenylethyl glucosinolate in oilseed rape (*Brassica napus* L.) leaves, while Mikkelsen *et al.* (2003) found that SA induced the accumulation of 4-methoxy-glucobrassicin (4IM) and reduced the contents of glucobrassicin (IM) and neoglucobrassicin (1IM). Moreover, Gigolashvili *et al.* (2008) observed that *MYB28*, the major regulator of aliphatic glucosinolate biosynthesis, was downregulated by SA treatment. The crosstalk between SA and JA in regulating glucosinolate biosynthesis has also been reported. It has been reported that high concentration of SA could inhibit the effect of JA, and others found that *Enterobacter radicincitans* DSM 16656 was able to induce priming via SA or JA signalling pathway to protect plants against potential pathogen attack (Brock *et al.*, 2013).

Sugars, generated by photosynthetic carbon fixation, play a central role in coordinating metabolic fluxes in response to the changing environment and in providing cells and tissues with necessary energy for continuing growth and survival (Wingler and Roitsch, 2008). Sugar sensing and signalling are highly complex, which impact many processes in plant growth and development (Rolland *et al.*, 2006). Sugar-induced glucosinolate accumulation has been observed in *Arabidopsis* and broccoli sprouts (Gigolashvili *et al.*, 2007; Guo *et al.*, 2011). In *Arabidopsis*, the content of glucosinolates is enhanced by glucose (Glu) and the expression level of *MYB28*, a transcription factor regulating aliphatic glucosinolate biosynthesis, is upregulated by application of Glu (Gigolashvili *et al.*, 2007). Besides, in broccoli sprouts, the expression of *Bo-Elong*, the major gene involved in aliphatic glucosinolate biosynthesis is also upregulated by sugars (Guo *et al.*, 2011). In the current study group’s previous work, ABI5 was shown to be the key regulator of glucose-induced aliphatic glucosinolate accumulation (Miao *et al.*, 2013).

Sugar can either positively or negatively impact on other signalling pathways including inorganic nutrients, phytohormones, and various stresses (Smeekens *et al.*, 2010). Sucrose has been shown to induce anthocyanin biosynthesis (Teng *et al.*, 2005). Loreti *et al.* (2008) demonstrated the crosstalk between hormone and sugar signalling in regulating anthocyanin biosynthesis. It was also reported that JA and sugars were two synergistic regulators of *vegetative storage proteins* (*VSP*) expression in soybean (*Glycine max* L.) (DeWald *et al.*, 1994). Little research focused on the interaction of SA and Glu is available, the only report is that the induction of pathogenesis-related (PR) protein-coding gene expression by sugar is almost completely inhibited in SA-deficient transgenic plant *NahG* (Thibaud *et al.*, 2004). Whether plant hormones and Glu interact in regulation of glucosinolate biosynthesis has not been reported so far. Being a rich source of glucosinolates, the model plant *A. thaliana* is a good system to elucidate the possible interactions between sugars and hormones in regulation of glucosinolate biosynthesis. This study investigated the interplay of JA/SA and Glu in inducing the accumulation of glucosinolates in *Arabidopsis*. Mutants related to JA and Glu signalling were used to clarify the possible mechanism underlying the crosstalk between JA and Glu in regulating glucosinolate accumulation in *Arabidopsis*.

## Materials and methods

### Plant materials and cultivation conditions

Seeds were sterilized in 75% ethanol for 30 s and then washed with distilled water five times, followed by sterilizing with 10% sodium hypochlorite for 3min and washing with distilled water five times. Subsequently, the seeds were rinsed in sterile water and incubated for 3 d at 4 °C. About 40 seeds were sown in a 100ml flask with 40ml low sugar growth medium containing Murashige-Skoog (MS) salt solution and 25mM Glu, and then shaken at 120rpm in plant growth chamber with a 16/8 light/dark cycle (110 µm photons m^–2^ s^–1^) at 22 °C and 70% relative humidity for 10 days.

Mutant seeds of *coronatine insensitive 1-2* (*coi1-2*) were generously provided by Dr Chuanyou Li (Institute of Botany, Chinese Academy of Sciences). The Glu-insensitive mutant *regulator of G-protein signalling 1-2* (*rgs1-2*) and *abscisic acid insensitive 5-7* (*abi5-7*) were obtained from Dr Jirong Huang (Shanghai Institute of Plant Physiology and Ecology, Chinese Academy of Sciences), and *salicylic acid induction deficient 2* (*sid2*) was provided by Dr Zhixiang Chen (Department of Botany and Plant Pathology, Purdue University). The *jasmonate insensitive 1* (*jin1/myc2*), *jasmonate resistant1-1* (*jar1-1*), and *constitutive expresser of PR genes 5-2* (*cpr5-2*) were provided by *Arabidopsis* Biological Resource Center (ABRC). The genetic background of all mutants was Columbia (Col-0).

### Plant hormones and Glu treatment

**Fig. 1.**
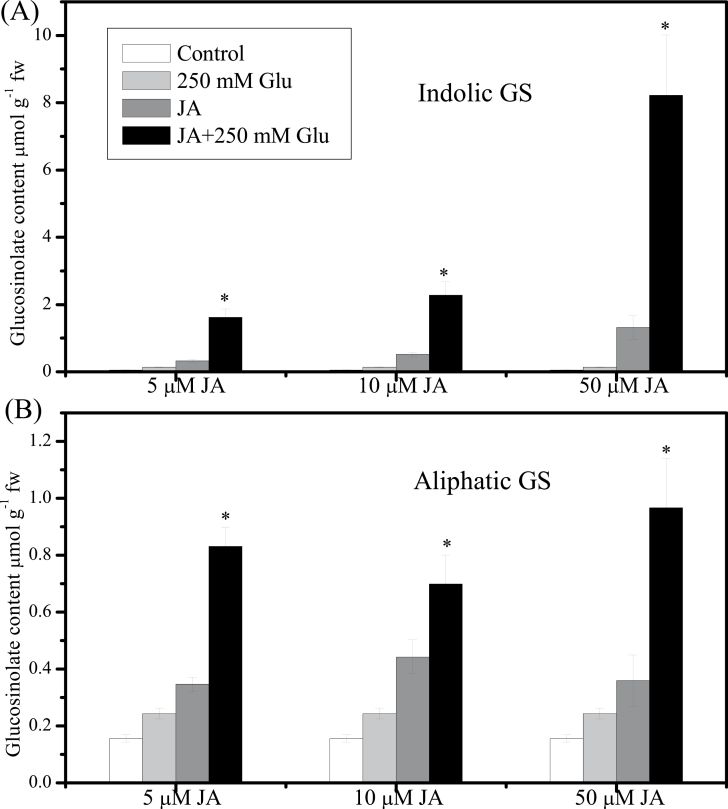
Effect of jasmonic acid (JA) or/and glucose (Glu) on glucosinolate (GS) contents. (A) Indolic GS level was measured in 13-d-old *Arabidopsis* seedlings treated with 5, 10, or 50 µM JA and/or 250mM Glu for 3 d. (B) Aliphatic GS level was measured in 13-d-old *Arabidopsis* seedlings treated with 5, 10, or 50 µM JA and/or 250mM Glu for 3 d. Control was treated with equal amount of water. Data are mean±standard error of five replicates per treatment. Values marked with an asterisk are significantly different for combined treatment of JA with Glu compared with all other treatments (*P* < 0.05).

Treatments were performed by adding JA/SA /Glu stock solutions to selected flasks and water to the control flasks. For Fig. 1, hormones and Glu were used at the following concentrations: JA: 5, 10, 50 μM; SA: 5, 10, 20, 50 μM; Glu: 250mM. For other figures, 5 μM JA and/or 250mM Glu were applied to 10-day-old seedlings for 3 d. The control was treated with water instead. For Fig. 7, 250 mM sorbitol was used as an osmotic control for glucose treatment. Seedlings were collected at 3 d after treatment with hormones and/or Glu for glucosinolate analysis. For each treatment, five replicates were taken for analysis.

### Glucosinolates assay

Glucosinolates were extracted and analysed as previously described with minor modifications (Guo *et al.*, 2011). Samples (200mg) were boiled in 1ml water for 10min. After transferring the supernatant to a clean tube, the residues were washed with water (1ml), and the combined aqueous extract was applied to a DEAE-Sephadex A-25 (35mg) column (pyridine acetate form) (Sigma, St Louis, MO, USA). Sinigrin (Sigma) was used as an internal standard for HPLC analysis. Desulphoglucosinolates were identified by comparison of retention time and quantified by peak area. The glucosinolate concentration was expressed as µmol g^–1^ fresh weight of *Arabidopsis* seedlings.

### RNA extraction

Ten-day-old seedlings treated with water, 250mM Glu, 5 μM JA, and 5 μM JA + 250mM Glu for 3 or 6h were collected for extraction of RNA. A total of 1ml of RNAiso Plus (Takara, Japan) was added to a cell pellet. After resuspending the cells, the tubes were vigorously shaken at room temperature for 1min. The samples were immediately added to 200 μl chloroform, shaken, and stored at room temperature for 5min; then the extraction mix was centrifuged for 15min at 12 000 *g* at 4 °C to promote phase separation. The aqueous phase was then retrieved and mixed with an equal volume of isopropanol, incubated at room temperature, and centrifuged to concentrate the precipitated RNA. The RNA pellet was washed using 75% ethanol, then air-dried, and finally dissolved in diethylpyrocarbonate water. Total RNA was isolated from *Arabidopsis* for two biological repeats using Trizol reagents according to manufacturer’s instruction (Takara).

### Reverse-transcription quantitative PCR

RNA samples were reverse-transcribed into cDNA using Prime Script RT Master Mix (Takara). The synthesized cDNAs were diluted 1/10 in H_2_O and their concentrations were normalized based on the amplification of *AtActin*. The reverse transcription PCR was performed with a total volume of 25 μl which contained 1µl diluted cDNA, 1 µl each 5 µM forward primer and reverse primer, 9.5 µl ddH_2_O, and 12.5 µl SYBR Green PCR Master Mix (Takara) on an ABI PRISM Step One Plus TM Real-Time PCR System. PCR amplification was performed using three-step cycling conditions of 95 °C for 30 s, followed by 40 cycles of 95 °C for 5 s and 58 °C for 1min. Expression level of *Arabidopsis ACTIN2* was used as an internal control and the expression of other genes was computed with the 2^–ΔΔCT^ method (Livak and Schmittgen, 2001). In order to allow an easier comparison of the effects of signals on the induction of genes related to glucosinolate biosynthesis, the expression level of the control was arbitrarily set to ‘1’. The primers used are listed in Supplementary Table S1 (available at *JXB* online).

### Statistical analysis

**Fig. 2.**
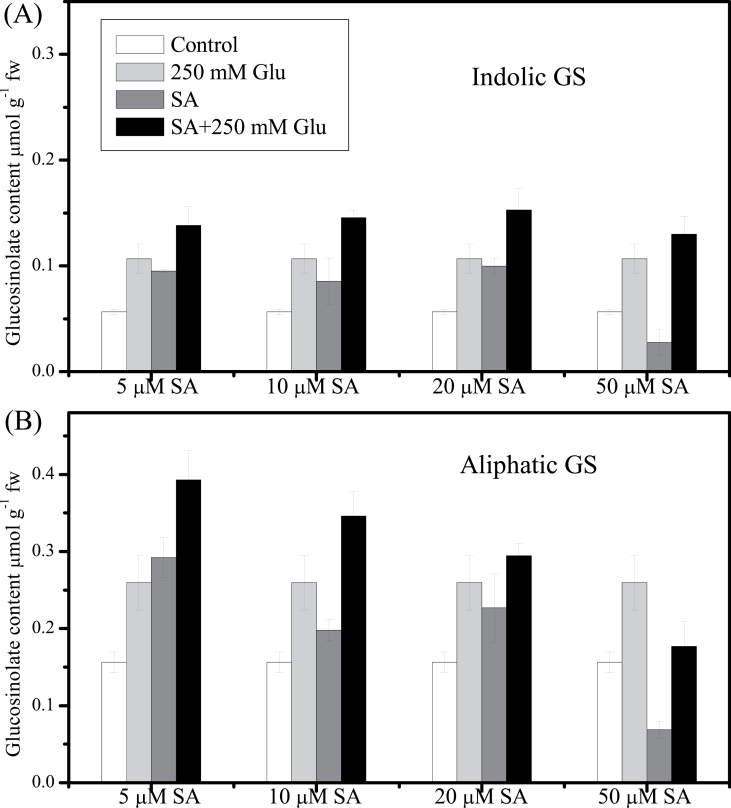
Effect of salicylic acid (SA) or/and glucose (Glu) on glucosinolate (GS) contents. (A) Indolic GS level was measured in 13-d-old *Arabidopsis* seedlings treated with 5, 10, 20, or 50 µM SA and/or 250mM Glu for 3 d. (B) Aliphatic GS level was measured in 13-d-old *Arabidopsis* seedlings treated with 5, 10, 20, or 50 µM SA and/or 250mM Glu for 3 d. Control was treated with equal amount of water. Data are mean±standard error of five replicates per treatment. Comparisons were made between the combined treatment of SA with Glu and the other treatments. There were no significant differences (*P* > 0.05).

**Fig. 3.**
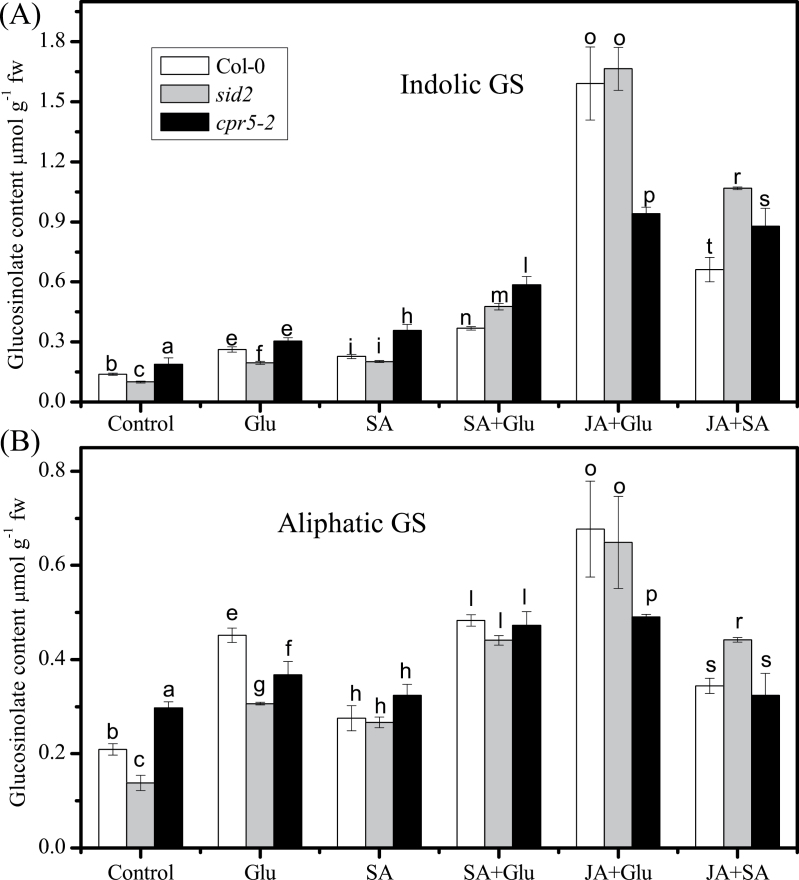
Effect of glucose (Glu) and combination of jasmonic acid (JA) and Glu on glucosinolate (GS) contents in SA mutants. (A) Indolic GS level was measured in 13-d-old seedlings of *sid2* (grey)*, cpr5-2* (black), and the wild type (Col-0; white) treated with 250mM Glu, 5 µM SA, 5 µM JA, 5 µM SA, or 5 µM JA/SA for 3 d. (B) Aliphatic GS level was measured in 13-d-old seedlings of *sid2* (grey)*, cpr5-2* (black), and the wild type (Col-0; white) treated with 250mM Glu, 5 µM SA, 5 µM JA, 5 µM SA, or 5 µM JA/SA for 3 d. Data are mean±standard error of five replicates per treatment. Values not sharing a common letter in a treatment group are significantly different (*P* < 0.05).

Statistical analysis was performed using SPSS version 11.5 (SPSS, Chicago, IL, USA). For Figs. 1–3 and 6–7, data was analysed by one-way ANOVA, followed by Turkey’s HSD multiple comparison test. For Fig. 5, data was analysed by one-way ANOVA. For Figs. 6 and 7, data was first analysed by one-way ANOVA, followed by Turkey’s HSD multiple comparison test for comparison of the effect of treatment on the same plant. Then data was analysed again using the independent-samples t-test for comparison of the effect of treatment on mutant and its corresponding wild type. The values were reported as means with their standard error for all results. Differences were considered significant at *P* < 0.05.

## Results

### Effect of JA and SA with or without Glu on accumulation of glucosinolates

*Arabidopsis* seedlings treated with low concentration (5 µM) of JA or SA showed enhanced accumulation of glucosinolates (Figs. 1 and 2). As shown in Fig. 1A and B, JA at 5 µM increased the cellular content of indolic glucosinolates by 6-times and that of aliphatic glucosinolates by 2-times compared with the control. When the concentration of JA was increased to 50 μM, the content of indolic glucosinolates increased up to 23-times of that in the control, but the growth of the seedlings was retarded (data not shown). In contrast, the effect of SA on glucosinolate content relied on the concentration of SA applied. Both indolic and aliphatic glucosinolates increased significantly after treatment with 5 μM SA, while treatment with SA at a high concentration (50 μM) significantly reduced the content of indolic and aliphatic glucosinolates when compared with the control (Fig. 2).

JA and Glu showed synergistic effect on inducing glucosinolate accumulation. As shown in Fig. 1, treatment with JA at concentrations of 5, 10, and 50 μM in combination with 250mM Glu significantly enhanced the accumulation of indolic and aliphatic glucosinolates compared with that treated with the corresponding concentration of JA or Glu alone. JA at the concentration of 5 μM was used in later experiments. In contrast, treatments with Glu (250mM) and SA (5, 10, 20, and 50 μM) only led to a slight increase in glucosinolate contents. The suppression of glucosinolate biosynthesis by 50 µM SA could be reversed by simultaneous Glu treatment (Fig. 2).

### Glucosinolate contents in SA-deficient and overproducing mutants

To confirm the effect of SA on glucosinolate accumulation, the SA-deficient mutant *sid2* and overproducing mutant *cpr5-2* were used. As shown in Fig. 3, both indolic and aliphatic glucosinolate contents decreased in *sid2* and increased in *cpr5-2* compared with the wild type. Glu promoted the accumulation of both indolic and aliphatic glucosinolates in *sid2, cpr5-2*, and Col-0. However, the accumulation of indolic and aliphatic glucosinolates was decreased in *cpr5-2* when compared with that in Col-0 after treated with JA and Glu, indicating that the synergistic effect of JA and Glu on glucosinolate accumulation is inhibited in *cpr5-2*.

### Synergistic effect of JA and Glu on induction of glucosinolate biosynthetic and regulatory genes

**Fig. 4.**
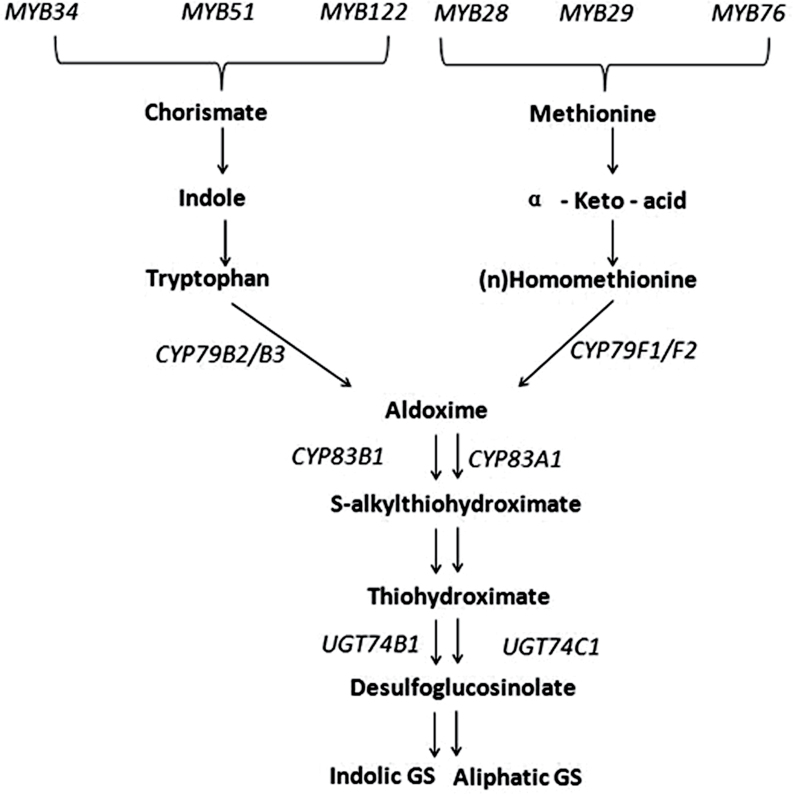
The biosynthetic pathway of indolic and aliphatic glucosinolate and the main genes involved in *Arabidopsis thaliana*.

To investigate whether JA-induced glucosinolate synthesis was associated with gene activation, this study analysed the expression pattern of genes involved in biosynthesis of glucosinolates (Fig. 4). Six MYB transcription factors, MYB28, MYB29, MYB76, MYB34, MYB51, and MYB122 are proved to regulate the biosynthesis of glucosinolates in *Arabidopsis* (Celenza *et al.*, 2005; Gigolashvili *et al.*, 2007, 2008).

**Fig. 5.**
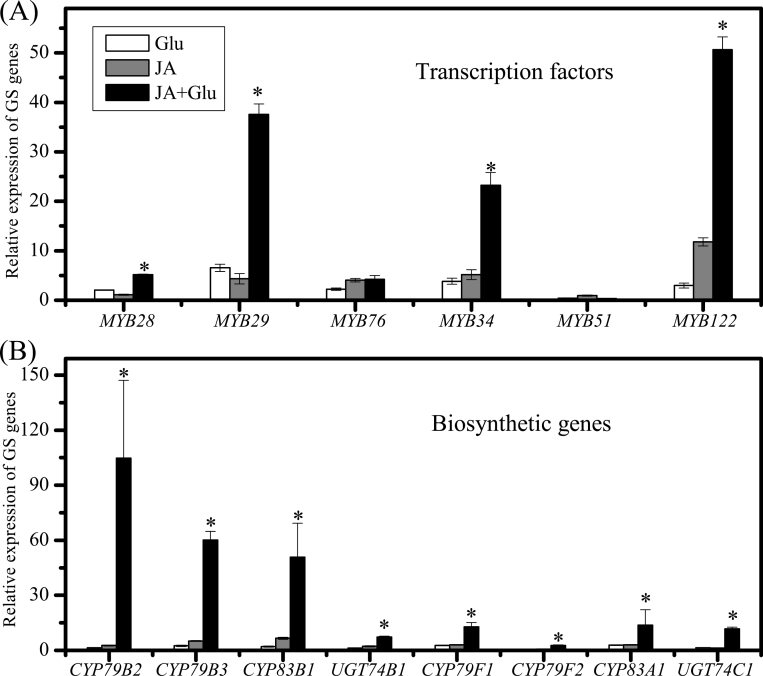
Effect of jasmonic acid (JA) or combination of JA and glucose (Glu) on expression of the genes involved in glucosinolate (GS) biosynthesis. (A) mRNA levels of *MYB28*, *MYB29*, and *MYB76* measured in 10-d-old seedlings treated with 5 µM JA or 5 µM JA and 250mM Glu for 3h. (B) mRNA levels of *CYP79B2*, *CYP79B3*, *CYP83B1*, *CYP79F1*, *CYP79F2*, *CYP83A1*, *UGT74B1*, and *UGT74C1* measured in 10-d-old seedlings treated with 5 µM JA or 5 µM JA and 250mM Glu for 6h. Relative expression level was measured by real-time PCR. Data are mean transcript level from two biological replicates. The expression level of the control samples was set to ‘1’.

As shown in Fig. 5A, the expression levels of *MYB28*, *MYB29*, *MYB76*, *MYB34*, and *MYB122* were increased by 5-, 38-, 4-, 23-, and 50-fold compared with the control, respectively, after being treated with JA together with Glu for 3 hours, while the expression levels of *MYB28*, *MYB29*, *MYB76*, *MYB34*, and *MYB122* after treatment with JA or Glu alone were all significantly increased by 1-, 3-, 3-, 4-, and 4-times and 2-, 7-, 2-, 4-, and 3-times, respectively.

The expression of the key biosynthetic genes such as *CYP79B2*, *CYP79B3*, *CYP79F1*, *CYP79F2*, *CYP83A1*, *CYP83B1*, *UGT74B1*, and *UGT74C1* was also detected in the present study (Fig. 5B). Among them, *CYP79B2*, *CYP79B3*, *CYP83B1*, and *UGT74B1* are related to the synthesis of indolic glucosinolate, while *CYP79F1*, *CYP79F2*, *CYP83A1*, and *UGT74C1* are involved in the biosynthesis of aliphatic glucosinolate. The results showed that the expression of all the glucosinolate biosynthetic genes tested was significantly higher under the combined treatment of JA and Glu in comparison with the treatment of JA or Glu alone.

### Glu-induced glucosinolate accumulation in JA signal transduction mutants

**Fig. 6.**
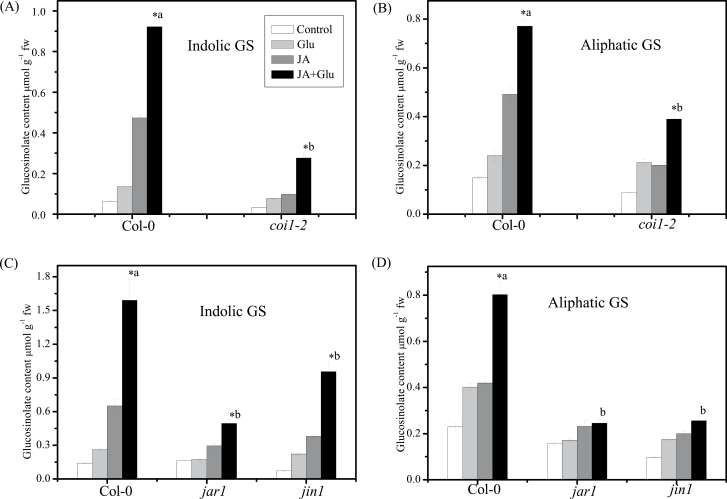
Effect of jasmonic acid (JA) and glucose (Glu) on glucosinolate (GS) accumulation in JA-signalling mutants *coi1-2*, *jin1*, and *jar1*. (A,B) Indolic GS accumulation (A) and aliphatic GS accumulation (B) in 13-d-old Col-0 and *coi1-2* seedlings on a low-Glu MS medium (control) or MS medium supplemented with 250mM Glu and/or 5 µM JA; data are mean of five replicates per treatment (mean ± standard error). (C, D) Indolic GS accumulation (C) and aliphatic GS accumulation (D) in 13-d-old Col-0, *jin1*, and *jar1* seedlings on a low-Glu MS medium (control) or MS medium supplemented with 250mM Glu and/or 5 µM JA; data are mean of five replicates per treatment (mean ± standard error). Values marked with an asterisk are significantly different for combined treatment of JA with Glu compared with all other treatments (*P* < 0.05). Values not sharing a common letter are significantly different from the corresponding wild type (*P* < 0.05).

To gain further insight into the possible interaction between JA and Glu in inducing glucosinolate accumulation, this study investigated the effect of JA signalling on glucosinolate accumulation in the presence/absence of Glu by using JA-insensitive mutants *coi1-2*, *jar1*, and *jin1*. In the JA signal transduction pathway, leucine-rich repeat protein COI1 is important for the perception of (+)-7-*iso*-jasmonoyl-isoleucine (JA-Ile) (Feys *et al.*, 1994; Sheard *et al.*, 2010). Mutation of *JASMONATE RESISTANT 1* (*JAR1*) reduces JA-Ile concentration (Staswick *et al.*, 1992), and JIN1/MYC2 is a basic helix–loop–helix transcription factor that activates the first wave of gene transcription upon jasmonate perception (Acosta and Farmer, 2010). The current study investigated the role of COI1, JAR1, and JIN1 in JA-dependent induction of glucosinolate accumulation. In Col-0, the contents of indolic and aliphatic glucosinolates were increased by 14- and 4-times, while only 8- and 4-times in *coi1*, 3- and 2-times in *jar1*, and 13- and 2-times in *jin1*, respectively, after the combined treatment of JA and Glu, indicating that induction of indolic and aliphatic glucosinolates in JA signalling mutants is reduced significantly compared with that in Col-0 (Fig. 6).

### Smaller synergistic effect of JA and Glu in the membrane receptor mutant *rgs1-2*

**Fig. 7.**
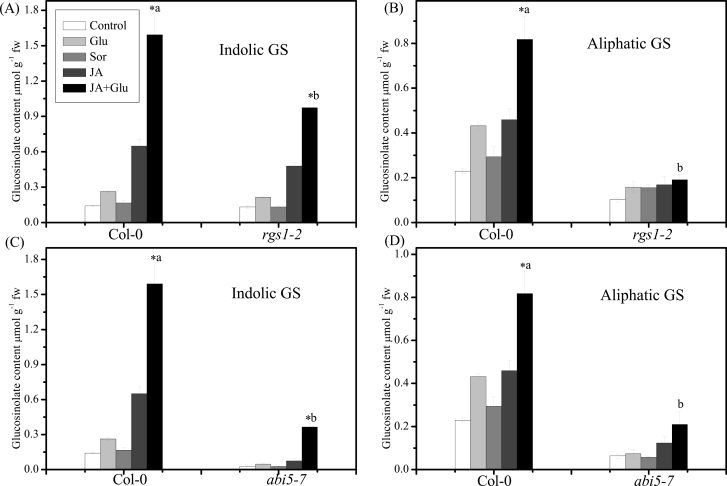
Role of RGS1 and ABI5 in the jasmonic-acid- (JA-) and glucose- (Glu-) induction of glucosinolate (GS) in *Arabidopsis*. (A, B) Indolic GS accumulation (A) and aliphatic GS content (B) in 13-d-old Col-0 and *rgs1-2* seedlings on a low-Glu MS medium (control) or MS medium supplemented with 250mM Glu, 250mM Sorbitol (Sor), 5 µM JA, and 250mM Glu + 5 µM JA; data are mean of five replicates per treatment (mean ± standard error). (C, D) Indolic GS accumulation (C) and aliphatic GS accumulation (D) in 13-d-old Col-0 and *abi5-7* seedlings on a low-Glu MS medium (control) or MS medium supplemented with 250mM Glu, 250mM Sorbitol (Sor), 5 µM JA and 250mM Glu+5 µM JA. Values marked with an asterisk are significantly different for combined treatment of JA with Glu compared with all other treatments (*P* < 0.05). Values not sharing a common letter are significantly different from the corresponding wild type (*P* < 0.05).

To identify the Glu signalling components involved in JA- and Glu-induced glucosinolate accumulation, this study analysed the response of Glu-insensitive mutant *rgs1-2* to Glu with or without JA. Lack of the G-protein-interacting membrane protein RGS1 impairs Glu sensing (Grigston *et al.*, 2008). The induction of glucosinolates, especially aliphatic ones, after JA or Glu treatment was significantly reduced in *rgs1-2* compared with the wild type (Fig. 7A, B). Moreover, the accumulation of glucosinolates was also reduced when treated with JA and Glu together, suggesting that the Glu membrane receptor RGS plays a key role in regulating glucosinolate accumulation by JA or Glu treatment or both.

### Glucosinolate accumulation in Glu-insensitive mutants

In addition to the membrane receptor mutant *rgs1-2*, other Glu signal transduction mutants, including *gin2-1* (mutation of *hexokinase1*, *HXK1*; Supplementary Fig. S3), *abi4-1* (Supplementary Fig. S4), and *abi5-7* (Fig. 7C, D) were also used to investigate whether they were involved in the inducing effect of combined JA and Glu treatment on glucosinolate biosynthesis. The results showed that the glucosinolate contents in the *abi5-7* mutant were significantly lower than those in the control (Fig. 7C, D). After treatment with JA or combination of JA and Glu, the seedlings of *abi5-7* mutant accumulated much less glucosinolates than those of the wild type, indicating that ABI5 plays an important role in inducing glucosinolate accumulation.

## Discussion

### Synergistic effect of JA and Glu on regulation of indolic and aliphatic glucosinolate accumulation

Little is known about the synergistic effect of JA and Glu in inducing glucosinolate biosynthesis, although the regulation of JA or methyl JA on indolic glucosinolate metabolism in *Brassica* species has been reported (Bodnaryk, 1994). The crosstalk between JA and sugar in regulation of anthocyanin biosynthesis has been investigated in *Arabidopsis*, which indicates that the induction of anthocyanins by JA is dependent on sucrose, and COI1 is the key component in inducing anthocyanins accumulation by JA (Loreti *et al.*, 2008). The current survey results suggested that JA exerts a synergistic effect with Glu on regulation of glucosinolate accumulation, and the inducing effect of JA on glucosinolate biosynthesis was independent of Glu (Fig. 1). The synergetic effect of JA and Glu was further supported by the finding of significantly elevated expression levels of the genes related to glucosinolate biosynthesis by the application of 5 μM JA and 250mM Glu together (Fig. 5), which indicated that JA and Glu regulate the accumulation of glucosinolates by activating the biosynthetic genes.

The effect of JA on indolic glucosinolate accumulation has been extensively reported. In low-sugar (25mM Glu) culture medium, application of 5 μM JA significantly enhanced the accumulation of indolic glucosinolates IM and 1IM (Supplementary Fig. S1). *MYB34* and *MYB122* were the most responsive transcription factors to JA treatment (Fig. 5A) and the accumulation of indolic glucosinolates, IM and 1IM, was enhanced by JA treatment (Supplementary Fig. S1A). Mikkelsen *et al.* (2003) also reported the induction of IM and 1IM by JA treatment, whereas Dombrecht *et al.* (2007) found that IM and 4IM were the JA-responsive indolic glucosinolates.

In addition to the induction of indolic glucosinolates, the application of JA also promoted the contents of aliphatic glucosinolates, including glucoiberin (S3), glucoraphanin (S4), and glucohirsutin (S8) (Supplementary Fig. S1B). This is consistent with the observations by Kliebenstein *et al.* (2002) and Mikkelsen *et al.* (2003) except for the enhancement of S4 by JA treatment. This could be further supported by the gene expression analysis. For instance, the expression levels of transcription factors *MYB29* and *MYB76* as well as synthetic genes *CYP79F1* and *CYP83A1* were all enhanced by JA treatment in the present study. Similar to the regulation of indolic glucosinolates, the synergistic effect of JA and Glu also existed in regulation of aliphatic glucosinolates. The expression levels of *MYB28* and *MYB29* as well as *CYP79B2*, *CYP79B3*, *CYP83B1*, and *UGT74B1* were all dramatically upregulated by JA plus Glu treatment. The inconsistency in JA-induced aliphatic glucosinolate profiles maybe due to the differences in cultivation conditions, concentrations of JA applied, and developmental stage of the materials used among different studies.

### Inhibiting role of SA at high concentration in glucosinolate biosynthesis

In several previous studies, SA was reported to play a positive role in accumulation of 4IM and 2-phenylethyl glucosinolates in *A. thaliana* and *B. napus*, respectively (Kiddle *et al.*, 1994; Brader *et al.*, 2001; Kliebenstein *et al.*, 2002). In contrast, SA was reported to suppress the glucosinolate synthesis and, sometimes, to have no effect (Mikkelsen *et al.*, 2003). The current study found that the effect of SA on glucosinolate biosynthesis was dependent on the concentration of SA applied (Fig. 2). Low concentration (5 μM) of SA enhanced the glucosinolate accumulation, while high concentration of SA (50 μM) decreased the contents of glucosinolate. This dosage-dependent effect of SA action was also observed in other SA-mediated regulation such as the expression of pathogenesis-related protein genes and the resistance to insects (Niki *et al.*, 1998; Anand *et al.*, 2003; Mur *et al.*, 2006). The role of JA in regulation of glucosinolate biosynthesis is different from that of SA in two aspects. Firstly, different kinds of glucosinolates are induced by JA and SA. JA mainly enhances the accumulation of IM and 1IM while 4IM is the major one responding to SA treatment. Secondly, the dosage response of JA and SA on glucosinolate accumulation is different. The levels of glucosinolate induction increase along with the increase of JA concentrations. In contrast, the effect of SA on glucosinolate biosynthesis is complicated and can either promote or inhibit glucosinolate accumulation depending on the SA concentration. Among the indolic glucosinolates in *Arabidopsis*, IM and 1IM are believed to function in the defence to herbivores, which are also frequently induced by JA. It is suggested that JA could boost the content of IM and 1IM to defend against the insect attack or other stresses. In addition, SA application specifically promoted the accumulation of one kind of indolic glucosinolates, 4IM, which is believed to be involved as a signalling molecule in, or a potential coactivator of, callose deposition (Clay *et al.*, 2009). Microbial pathogens can also trigger callose formation in *Arabidopsis* leaves via SA-dependent pathway (DebRoy *et al.*, 2004). Moreover, after infection with *Blumeria graminis*, plants accumulate high levels of 4IM, which makes 4IM a critical part in defence to fungal infection (Bednarek *et al.*, 2011). Therefore, it is possible that SA could be involved in defence partly through regulating the metabolism of indolic glucosinolates, especially 4IM. However, the regulation mechanism of SA on 4IM and its hydrolysis products is still unknown.

Application of SA with Glu showed additive effects on glucosinolate accumulation (Fig. 2). This was further demonstrated by analysis of the glucosinolate contents in SA-deficient *sid2* and SA-overproducing *cpr5-2* mutants treated with Glu. The different effect between SA and JA with Glu might be due to the difference in the composition and contents of glucosinolate induced by these two signal molecules. Treatment with SA + Glu mainly enhanced the content of 4IM, while JA+ Glu increased the accumulation of IM and 1IM substantially (Supplementary Figs. S1A and 2A). The decrease in endogenous SA content had no effect on JA-Glu-induced accumulation of glucosinolates. However, the overproduction of SA negatively regulated the JA-Glu-induced glucosinolate biosynthesis. This is in accordance with the repressing role of SA at an excessive dose in JA induced glucosinolate accumulation (Mikkelsen *et al.* 2003). In the current work, the accumulation of glucosinolates was increased in *cpr5-2* itself under low-sugar cultivated condition, but when it was subjected to Glu or combination of JA and Glu treatment, the inhibitory effect of high SA content emerged. A possible explanation is that the balance between SA and JA signalling is needed for the regulation of glucosinolate accumulation by Glu.

### *COI1*, *JAR1*, and *JIN1* are involved in the regulation of glucosinolate accumulation by JA plus Glu treatement

Mikkelsen *et al.* (2003) demonstrated that COI1 is necessary for the regulation of glucosinolate biosynthesis by JA in *Arabidopsis*. Genome-wide transcriptional profiling of wild type and jasmonate signal-transduction mutant *jasmonate insensitive 1* (*jin1/myc2)* plants followed by functional analysis has revealed that MYC2 negatively regulates tryptophan-derived secondary metabolism such as indolic glucosinolate biosynthesis during jasmonate signalling (Dombrecht *et al.*, 2007). Two MYB transcription factors, MYB29 and MYB34, specifically regulate several genes related to the biosynthesis of aliphatic and indolic glucosinolates, respectively, and participate in the jasmonate-mediated induction of glucosinolate biosynthesis (Hirai *et al.*, 2007). In the present study, the deficiency of JIN1 and JAR1 as well as COI1 led to significant decrease in glucosinolate contents compared with their corresponding wild type after treatment with JA and Glu together (Fig. 6), indicating an important role of COI1, JAR1, and JIN1 in regulation of glucosinolate biosynthesis by JA and Glu.

### RGS1 and ABI5 are needed to regulate glucosinolate biosynthesis by JA + Glu

Glu was found to regulate plant metabolism as a signal (Rolland *et al.*, 2006; Smeekens *et al.*, 2010). In addition, glucose can affect the accumulation of glucosinolates as a substrate. In the biosynthesis of glucosinolate core structure, the intermediate product thiohydroximate was glycosylated by *S*-glucosyltransferases (S-GT). The distinct effect of Glu and sorbitol treatment on indolic and aliphatic glucosinolate accumulation indicated that Glu might function as a signal in inducing glucosinolate biosynthesis (Fig. 7). RGS1, the G-protein-interacting membrane protein, was the putative membrane receptor of Glu signalling in plants (Grigston *et al.*, 2008). Significant decrease in the content of both indolic and aliphatic glucosinolates in *rgs1-2* mutant after treatment with JA plus Glu indicates that RGS1 plays an important role in regulation of glucosinolate accumulation by JA and Glu. Previous work indicated that glucose-induced aliphatic glucosinolate biosynthesis is regulated by HXK1- and/or RGS1-mediated signalling via transcription factors MYB28, MYB29, and ABI5 (Miao *et al.*, 2013). ABI5 encodes a transcription factor belonging to a large basic leucine zipper gene family, which is a putative glucose signalling component downstream of HXK1 (Ramon *et al.*, 2008). It was also reported to be a vital factor in glucose-induced aliphatic glucosinolate accumulation. In mutant *abi5-7* treated with glucose, the expression of transcriptional factors *MYB28*, *MYB29*, and *MYB76* was all downregulated significantly compared with that in Col-0 (Miao *et al.*, 2013). This observation suggested that ABI5 might modulate glucosinolate biosynthesis via interaction with MYB28, MYB29, or MYB76. Among the Glu signalling mutants (*gin2-1*, *abi4-1*, and *abi5-7*) tested in this study (Supplementary Figs. S3 and S4), *abi5-7* was the only one with significantly decreased glucosinolate contents before and after treatment with JA and Glu treatment when compared with the wild type (Fig. 7C, D).

**Fig. 8.**
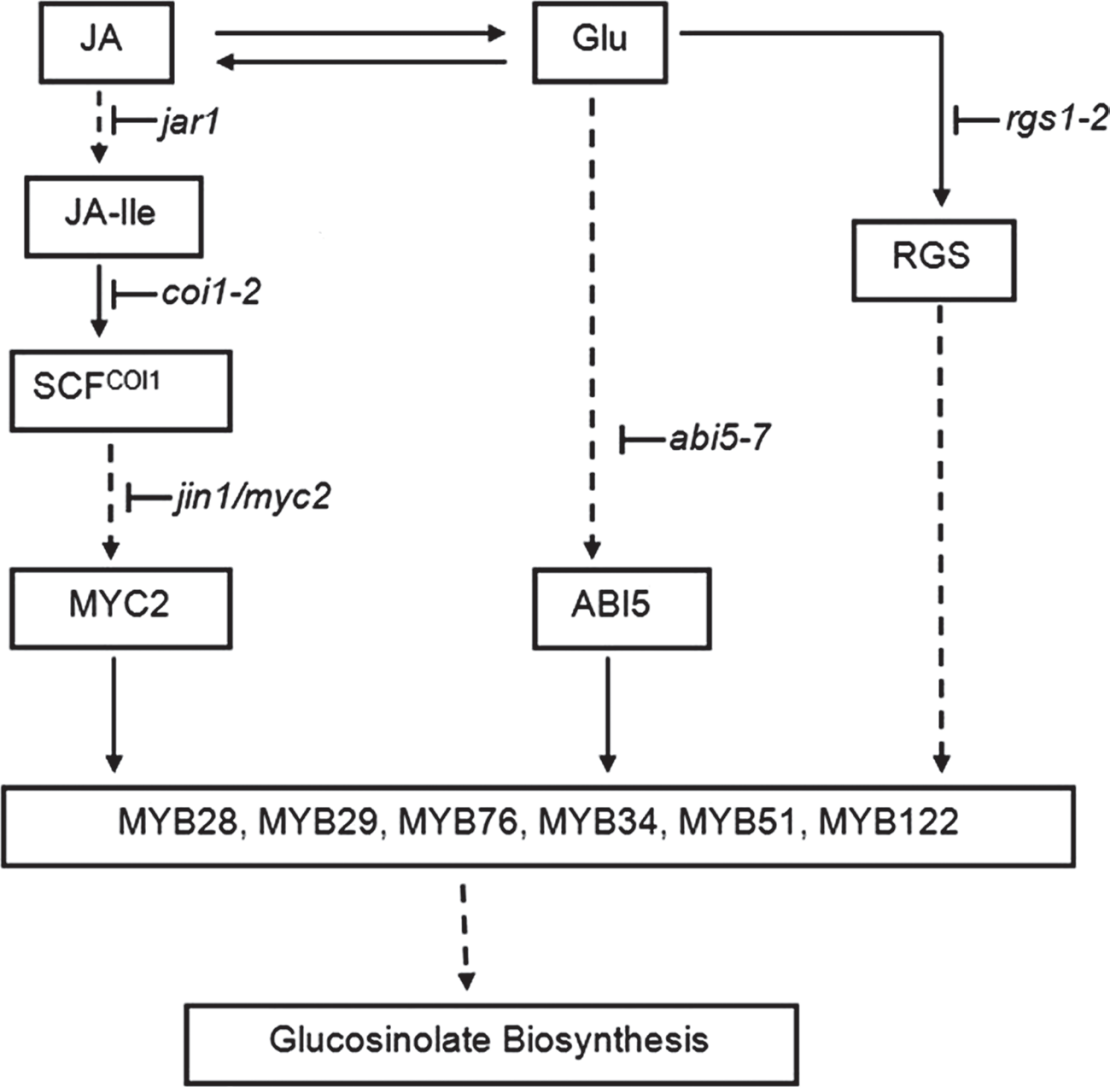
The possible regulation mechanism of jasmonic acid (JA) and glucose (Glu) on glucosinolate biosynthesis in *Arabidopsis*. JA and Glu synergistically modulate the accumulation of glucosinolates. The JA signalling components JAR1, COI1, and JIN1 are necessary for the regulation of glucosinolate biosynthesis by JA and/or Glu treatments. RGS1, the putative membrane receptor of glucose signalling, plays a key role in the induction of glucosinolate accumulation by JA and Glu, and transcription factor ABI5 is involved in the synergetic effect of the JA and Glu signalling pathways in the regulation of glucosinolate biosynthesis. JA-Ile, (+)-7-*iso*-jasmonoyl-isoleucine; SCF, Skp/Cullin/F-box. 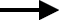 activation; 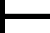, inhibition; 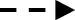, multistep reaction.

In conclusion, Glu-induced glucosinolate biosynthesis was enhanced by the addition of JA via enhancing the expression of the biosynthetic genes. The JA signalling components COI1, JAR1, and JIN1 are necessary for the regulation of glucosinolate biosynthesis by JA and/or Glu treatments. RGS1, the putative membrane receptor of glucose signalling plays a key role in the induction of glucosinolate accumulation by JA and Glu, and transcription factor ABI5 is involved in the convergence of the JA and Glu signalling pathways in the regulation of glucosinolate biosynthesis (Fig. 8). These findings contribute to the understanding of the regulatory network of glucosinolate metabolism by signalling molecules.

## Supplementary material

Supplementary data are available at *JXB* online.

Supplementary Table S1. Primers used in reverse-transcription quantitative PCR.

Supplementary Fig. S1. Effect of 5 µM JA and/or 250mM glucose on individual indolic and aliphatic glucosinolate biosynthesis.

Supplementary Fig. S2. Effect of 5 µM JA and/or 250mM glucose on individual indolic and aliphatic glucosinolate biosynthesis.

Supplementary Fig. S3. Role of HXK in the jasmonic-acid and glucose induction of glucosinolate accumulation in *Arabidopsis*.

Supplementary Fig. S4. Role of ABI4 in the induction of jasmonic acid and glucose on glucosinolate accumulation in *Arabidopsis*.

Supplementary Data
