# Supplementary material for: Jasmonic acid and glucose synergistically modulate the accumulation of glucosinolates in Arabidopsis thaliana
Source: J Exp Bot. 2013 Oct 22;64(18):5707–19. doi: 10.1093/jxb/ert348 (PMC3871825; doi:10.1093/jxb/ert348)
Supplement: Supplementary Data [file supp_64_18_5707__index.html]

Jasmonic acid and glucose synergistically modulate the accumulation of glucosinolates in Arabidopsis thaliana — Jasmonic acid and glucose synergistically modulate the accumulation of glucosinolates in Arabidopsis thaliana — Supplementary Data 

# Jasmonic acid and glucose synergistically modulate the accumulation of glucosinolates in *Arabidopsis thaliana*

## Supplementary Data

Data files

**Files in this Data Supplement:**

- Supplementary Data - Supplementary Data
