# Supplementary material for: Jasmonic acid and glucose synergistically modulate the accumulation of glucosinolates in Arabidopsis thaliana
Source: J Exp Bot. 2013 Oct 22;64(18):5707–19. doi: 10.1093/jxb/ert348 (PMC3871825; doi:10.1093/jxb/ert348)
Supplement: Supplementary Data [file supp_ert348_jexbot098103_file001.pdf]

Jasmonic acid and glucose synergistically modulate the accumulation of glucosinolates in *Arabidopsis thaliana*

Rongfang Guo[1], Wangshu Shen[1], Hongmei Qian[1], Min Zhang[1], Lihong Liu [1], Qiaomei Wang[1, 2]*

Supplementary materials

Table S1 Primers used in reverse transcription-quantitative polymerase chain reaction (RT-qPCR) analysis.

Gene Name	Locus Code	Primes sequence (5'-3') for RT-PCR
MYB34	At5g60890	CGGGTCTTAAGTAATTAGCC<br>AAGAAAGGAGCTTGGACTCC
MYB51	At1g18570	ACAAATGGTCTGCTATAGCT<br>CTTGTGTGTAACTGGATCAA
MYB122	At1g74080	TCCGTTGAGTCTTGTTTGGA<br>TTGTCAATCCCTTCACAGGA
MYB28	At5g61420	TCCCTGACAAATACTCTTGCTGAT<br>CATTGTGGTTATCTCCTCCGAATT
MYB29	At5g07690	CAATACTGGAGGAGGATATAACC<br>AGTTCTTGTCGTCATAATCTTGG
MYB76	At5g07700	ACGTTTAATCGATGATGGCA<br>ATGGGCTCAACTGGATTAGG
CYP79F1	At1g16410	CCATACCCTTTTCACATCCTACTGTCT<br>GTAGATTGCCGAGGATGGGC
CYP79F2	At1g16400	ACTAGGATTTATCGTCTTCATCGCA<br>CTAGGACGAGTCATGATTAGTTCGG
CYP79B2	At4g39950	TTTGATGGATTGTCTGGCGC<br>CAAAGACGAACAAGGCAACC
CYP79B3	At2g22330	CGGTTTGTTTATCATCTCCGC<br>TTGCTTACCGCTGATGAAATC
CYP83A1	At4g13770	TTCAAGAGGTTGTCAATGAGACGC<br>CTACAATATCCAAGATGACGGCTTT
CYP83B1	At4g31500	TCCGACCTTTTCCCTTATTTCG<br>TTGAGACGTGCACTGAGACCAG
UGT74B1	At1g24100	TATCTTGATGATCGGATGGA<br>TACTGACTGAGCCTGCTTAG
UGT74C1	At2g31790	GGTGGTTCCATCGAAGTTCT<br>TGTTTCCCAACCACTTCAAA
Actin2	At3g18780	TAACTCTCCCGCTATGTATGTCGC<br>CCACTGAGCACAATGTTACCGTAC

Figure S1 Effect of jasmonic acid (JA) and/or glucose (Glu) on individual indolic and aliphatic glucosinolate (GS) biosynthesis. (A) Individual indolic GS level was measured in 13-d-old *Arabidopsis* seedlings treated with 5 µM JA and/or 250 mM Glu for 3 d. (B) Individual aliphatic GS level was measured in 13-d-old *Arabidopsis* seedlings treated with 5 µM JA and/or 250 mM Glu for 3 d. Each data is the mean of five replicates per treatment (mean ± standard error). Values not sharing a common letter are significantly different at $p < 0.05$.

Figure S2 Effect of salicylic acid (SA) and/or glucose (Glu) on individual indolic and aliphatic glucosinolate (GS) biosynthesis. (A) Individual indolic GS level was measured in 13-d-old *Arabidopsis* seedlings treated with 5 µM SA and/or 250 mM Glu for 3 d. (B) Individual aliphatic GS level was measured in 13-d-old *Arabidopsis* seedlings treated with 5 µM SA and/or 250 mM Glu for 3 d. Each data is the mean of five replicates per treatment (mean ± standard error). Values not sharing a common letter are significantly different at $p < 0.05$.

Figure S3 Role of HXK in the jasmonic-acid- (JA-) and glucose- (Glu-) induction of glucosinolate (GS) in *Arabidopsis*. (A) Indolic GS accumulation in 13-d-old Col-0 (open bars) and *gin2-1* (closed bars) seedlings on a low-Glu MS medium (Control) or MS medium supplemented with 250 mM Glu, 250 mM Sorbitol (Sor), 5 µM JA and 250 mM Glu+5 µM JA. (B) Aliphatic GS content in 13-d-old Col-0 (open bars) and *gin2-1* (closed bars) seedlings on a low-Glu MS medium (Control) or MS medium supplemented with 250 mM Glu, 250 mM Sor, 5 µM JA and 250 mM Glu+5 µM JA. Each data is the mean of five replicates per treatment (mean ± standard error). Values marked with an asterisk are significantly different at $p < 0.05$ among the combined treatment of JA together with Glu and other treatments (Control, Glu, Sor and JA). Values not sharing a common letter are significantly different at $p < 0.05$ with the corresponding wild type under application of JA and Glu together.

Figure S4 Role of ABI4 in the induction of jasmonic acid (JA) and glucose (Glu) on glucosinolate (GS) accumulation in *Arabidopsis*. (A) Indolic GS accumulation in 13-d-old Col-0 (open bars) and *abi4-1* (closed bars) seedlings on a low-Glu MS medium (Control) or MS medium supplemented with 250 mM Glu, 250 mM Sorbitol (Sor), 5 µM JA and 250 mM Glu+5 µM JA. (B) Aliphatic GS accumulation in 13-d-old Col-0 (open bars) and *abi4-1* (closed bars) seedlings on a low-Glu MS medium (Control) or MS medium supplemented with 250 mM Glu, 250 mM Sor, 5 µM JA and 250 mM Glu+5 µM JA. Values marked with an asterisk are significantly different at $p < 0.05$ among the combined treatment of JA together with Glu and other treatments (Control, Glu, Sor and JA). Values not sharing a common letter are significantly different at $p < 0.05$ with the corresponding wild type under application of JA and Glu together.

Figure S1

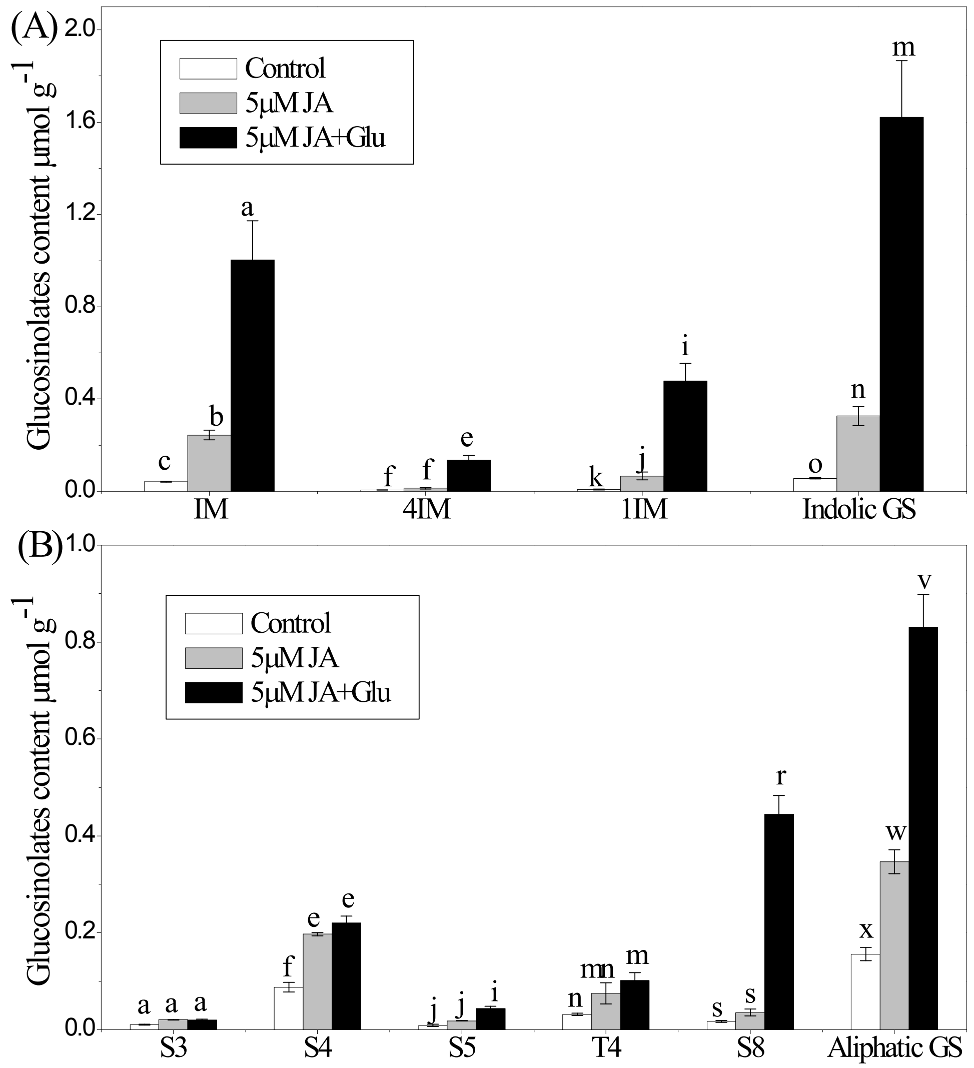

(A)
Control
5μM JA
5μM JA+Glu
Glucosinolates content μmol g-1
2.0
1.6
1.2
0.8
0.4
0.0
IM
4IM
1IM
Indolic GS
(B)
1.0
0.8
0.6
0.4
0.2
0.0
S3
S4
S5
T4
S8
Aliphatic GS

Figure S2

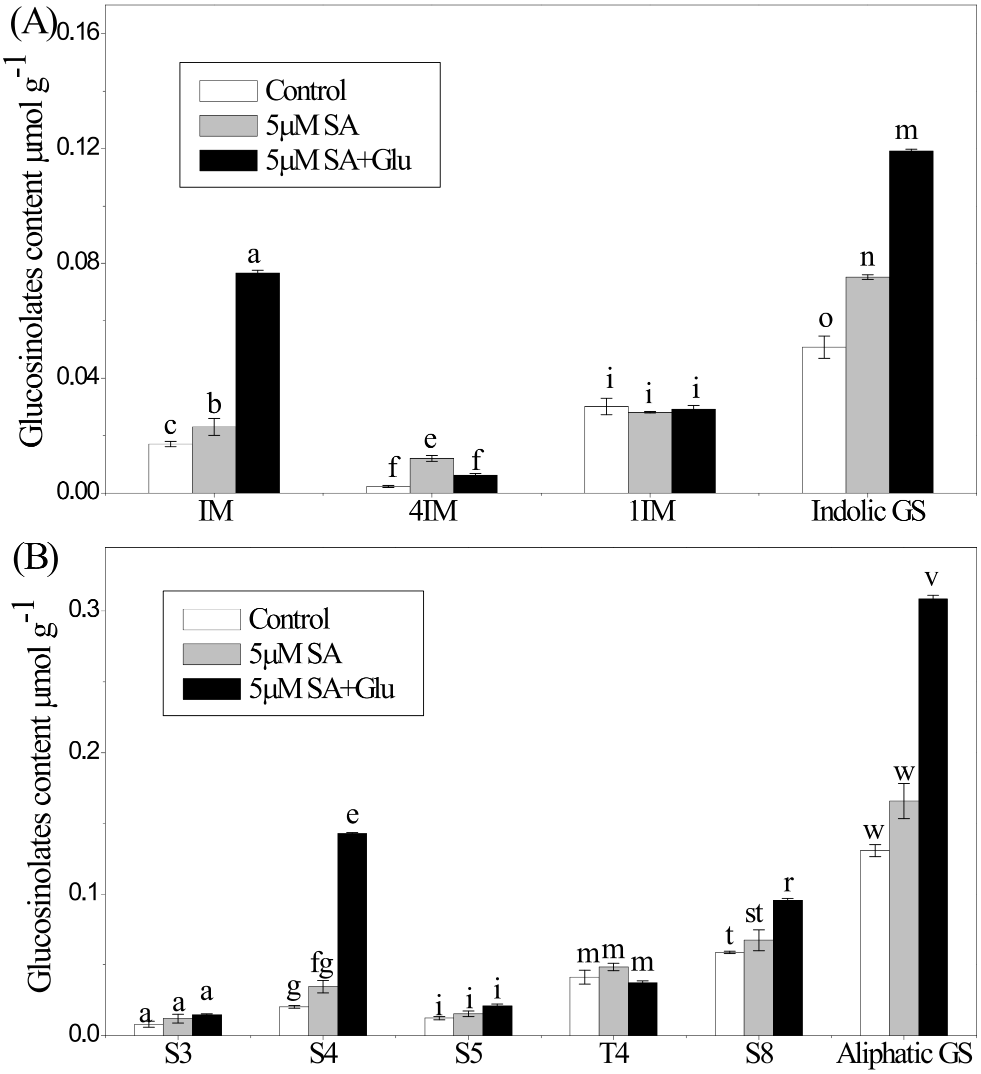
(A)
Glucosinolates content µmol g-1
0.00
0.04
0.08
0.12
0.16
Control
5µM SA
5µM SA+Glu
c
b
a
f
e
f
i
i
i
o
n
m
IM
4IM
1IM
Indolic GS
(B)
Glucosinolates content µmol g-1
0.0
0.1
0.2
0.3
Control
5µM SA
5µM SA+Glu
a
a
a
g
fg
e
i
i
i
m
m
m
t
st
r
w
w
v
S3
S4
S5
T4
S8
Aliphatic GS

Figure S3

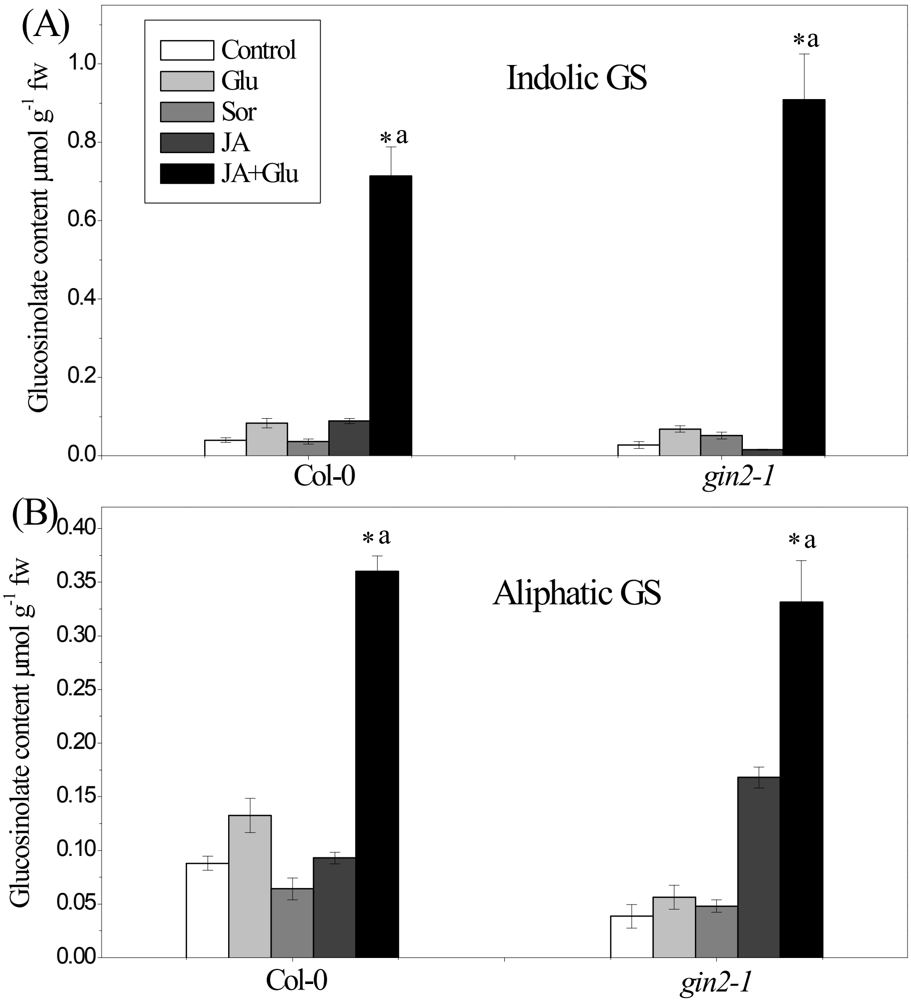
(A)
Control
Glu
Sor
JA
JA+Glu
Indolic GS
Glucosinolate content µmol g-1 fw
1.0
0.8
0.6
0.4
0.2
0.0
*a
*a
Col-0
gin2-1
(B)
Aliphatic GS
Glucosinolate content µmol g-1 fw
0.40
0.35
0.30
0.25
0.20
0.15
0.10
0.05
0.00
*a
*a
Col-0
gin2-1

Figure S4

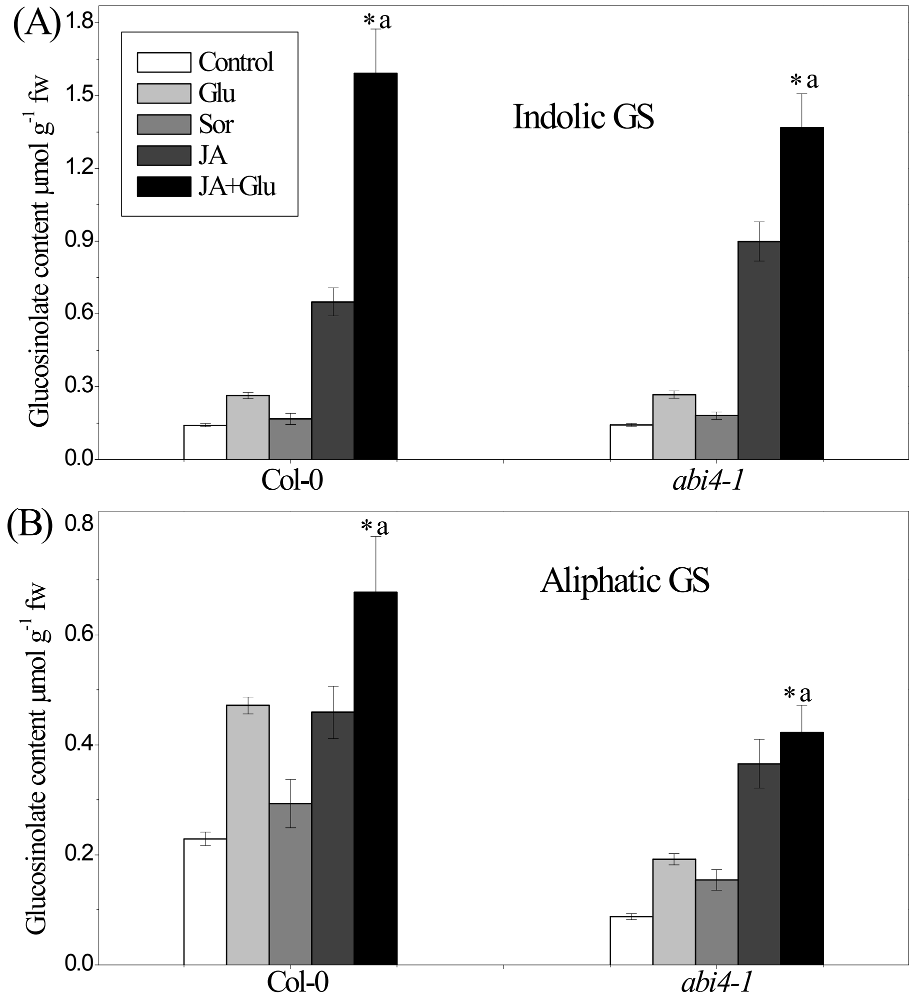
(A)
Glucosinolate content µmol g-1 fw
1.8
1.5
1.2
0.9
0.6
0.3
0.0
Control
Glu
Sor
JA
JA+Glu
Indolic GS
*a
*a
Col-0
abi4-1
(B)
Glucosinolate content µmol g-1 fw
0.8
0.6
0.4
0.2
0.0
Aliphatic GS
*a
*a
Col-0
abi4-1
